# How Healthcare Professionals Perceive Emergency Pediatric Care Provision in Two Public Hospitals in Greece: A Cross-Sectional Study

**DOI:** 10.3390/pediatric18010027

**Published:** 2026-02-05

**Authors:** Eleni Vathi, Konstantinos Petsios, Evangelos Dousis, Ioannis Koutelekos, Despoina Koumpagioti, Eirini Anastasopoulou, Anastasia Ntikoudi, Eugenia Vlachou, Eleni Evangelou

**Affiliations:** 1 Department of Nursing, University of West Attica, 12243 Athens, Greece; evathi@uniwa.gr (E.V.); edousis@uniwa.gr (E.D.); ikoutel@uniwa.gr (I.K.); dkoumpagioti@uniwa.gr (D.K.); antikoudi@uniwa.gr (A.N.); evlachou@uniwa.gr (E.V.); elevagel@uniwa.gr (E.E.); 2 Faculty of Nursing, National and Kapodistrian University of Athens, 11527 Athens, Greece; 3 General and Oncological Public Hospital Kifisias “Ag. Anargyroi”, 14564 Athens, Greece; eanastasopoulou@uniwa.gr

**Keywords:** pediatric emergency care, quality of health care, family-centered care, health personnel, professional practice gaps

## Abstract

**Background/Objectives**: High-quality pediatric emergency care requires timely access, effective communication, privacy, pain management, comfort, and child- and family-centered practices; however, implementation may be constrained by several barriers. The aim of the study was to evaluate the quality of pediatric emergency care as perceived by healthcare professionals, with emphasis on child-centered care and variations based on workplace and professional characteristics. **Methods**: A cross-sectional survey was performed in the emergency departments in two tertiary public pediatric hospitals in Athens, Greece. A study-developed 14-item Quality of Care Assessment Scale with paired ratings of agreement with quality principles and implementation in practice was completed by 162 professionals (122 doctors, 24 nurses, 16 assistant nurses). Independent items evaluated perceived barriers, overall assessments (0–100), and information provided to parents/children (5-point Likert scale). Inferential tests and descriptive statistics were also used (*p* < 0.05). **Results**: There was a significant degree of agreement with quality principles, but there was a constant lack of implementation (principle–practice gap). The primary perceived weakness was waiting times; child-friendly settings and privacy during examinations and information-giving were also lacking. Internal consistency ranged from good to acceptable (implementation α = 0.800; agreement α = 0.711). Children were most frequently rated as “moderately informed” (48.1%), while parents were most frequently rated as “quite informed” (50.0%). Compared to the organization of care (mean 60.85), perceived safety was higher (mean 73.27). Perceptions varied by age, educational level, profession, department, shift rotations, and hospital. The main barriers were workload (30.2%), poor coordination (34.0%), and lack of resources (46.9%). **Conclusions**: Health professionals seem to perceive that consistent delivery of child-centered care is impaired by organizational and structural limitations. Reducing the standards-to-practice gap requires targeted system-level interventions that focus on staffing, care organization, environment, and professional support.

## 1. Introduction

The quality of the provided care stands as a key priority in the current pediatric care and health systems around the world, and a wide set of indicators have been employed to assess and systematically monitor quality of hospital care for children [1]. Health managers invest more in services that achieve the best clinical performance in combination with high satisfaction from their users [2]. Care quality directly impacts children’s immediate clinical outcomes, their long-term health paths, and family trust in healthcare services, and quality improvement processes can significantly address the gaps in health services [3].

In emergency and outpatient pediatric settings, quality involves more than just getting the diagnosis right and providing effective treatment. Evidence shows that there is a positive connection between child and caregiver experience, clinical effectiveness, and patient safety, highlighting that “quality” should be addressed as a multidimensional concept rather than a purely clinical outcome [2,3]. In addition, timely access, clear communication, respect for dignity, protection of privacy, pain management, and comfort are core components of high-quality care and consistent with family- and child-centered care principles [1,4]. In pediatric emergency contexts specifically, family caregivers commonly report emotional support and communication as critical needs, reinforcing the central role of interpersonal care processes in overall quality and satisfaction [5].

Emergency departments are amongst the most challenging environments for providing pediatric care. Recent research finding indicate that crowding, staffing shortages, and higher service demand impact waiting times, continuity of care, and clinicians’ ability to address children’s emotional and psychosocial needs along with caregivers’ emotional and communication needs and can lead to less than optimal healthcare provision [5,6]. These issues are especially significant in public hospital systems, where limited resources can hinder the consistent application of best practices in communication, pain prevention, and family involvement [6]. Evidence also shows that structured interventions aimed at strengthening patient and family involvement in escalation of care can improve safety and responsiveness across community and hospital settings, highlighting the importance of meaningful family engagement even in high-pressure environments such as emergency care [7].

The views of healthcare professionals are important for a holistic assessment of the quality of the provided pediatric care, since they add a distinct perspective shaped by day-to-day clinical practice, organizational constraints, interprofessional collaboration, and ethical obligations toward children [8,9]. While experiences and preferences reported by children and caregivers are invaluable, health professionals provide a different perspective, highlighting how quality of care is influenced by staffing levels, access to training, and workplace culture [10].

The concept of family- and child-centered care in pediatric emergency settings describes how care should currently be delivered. It emphasizes working in partnership with families, placing the child and their family at the center of healthcare planning and delivery [11,12]. This approach focuses on respect, sharing information, encouraging participation, and promoting collaboration. The care provided should connect with the family’s priorities and values [11,12,13]. Child-centered care specifically highlights the child’s rights, voice, and developmental needs, and recognizes the child as an individual and active collaborator [11]. The concept of child- and family-centered care guides the provision of care through respectful partnership with the family, using communication, participation, and collaboration to tailor decisions and support to the child’s and family’s context, with increasing emphasis on addressing equity-related barriers that shape families’ ability to engage in care [12,13,14].

At the same time, the literature defines the standardization of pediatric emergency care as “pediatric readiness” [15]. This means ensuring that every emergency department has equipment and medication systems specifically for children, as well as trained and competent staff [15,16]. It also includes having written policies and protocols, quality improvement processes, and designated pediatric emergency care coordination [16,17]. This framework helps ensure that the system consistently delivers safe, high-quality care for children in any setting [15,16,17,18].

Public pediatric hospitals are essential for providing outpatient and emergency care in Greece, with dedicated facilities and specialized staff (24/7). In Greece, pediatric emergency care is mainly provided in public pediatric hospitals since they offer services for free and there is no direct cost to the patient. Following the economic crisis, there has been an increase of 20% in the attendance rate, with a mean age of 6.1 ± 4 years in 2017 [19]. In Attica, there are three main public hospitals, with an overall total of 185.000–200.000 annual visits to their emergency departments [20]. However, the perspectives of health professionals regarding the standard of pediatric emergency care are not well documented. It is necessary to examine their viewpoints in order to identify organizational and structural gaps, set priorities for quality improvement initiatives, and develop policies that conform to contemporary international standards. Therefore, the purpose of this study was to assess how healthcare professionals perceive the quality of pediatric emergency and outpatient care in two major public children’s hospitals in Athens, with an emphasis on key components of family- and child-centered care and how these perceptions differ depending on professional and workplace characteristics.

## 2. Materials and Methods

### 2.1. Study Design and Setting

This cross-sectional, descriptive survey investigated the quality of emergency pediatric care as viewed by healthcare professionals working in two major pediatric public hospitals located in Athens, Greece (Pentelis General Children’s Hospital and Panagiotis & Aglaia Kyriakou Children’s Hospital). The two hospitals are tertiary referral centers of the Greek National Health System, offering specialized pediatric emergency and outpatient services for a large volume of pediatric patients, and are representative centers of public pediatric healthcare in Greece. This study aims to identify key factors influencing the quality of the provided care as well as areas for improvement in the organization and delivery of pediatric emergency services, as assessed by the healthcare professionals working in these settings. The preliminary results of this study are presented along with data regarding the development of the instrument.

### 2.2. Data Collection Instrument

A structured questionnaire was employed to collect data on the following:

- Demographic information (12 items).
- Health Professionals’ Quality of Care Assessment Scale (14 double items—level of agreement and implementation in clinical practice) assessing key aspects of patient and parental experience including accessibility to care, respect, child-friendliness, communication and information continuity, pain management, and respect for privacy.

The steps for the development of the scale were as follows [21,22,23]:

- Literature review and expert panel (content validity), which resulted in 16 items. The content validity of the questionnaire was established through a panel of experts, including pediatricians, nurses, nursing academics, and healthcare quality specialists, who reviewed the items for clarity, relevance, and cultural appropriateness to the Greek healthcare setting.
- Cognitive interviews (2 pediatricians, 2 nurses, 1 assistant nurse), which resulted in the merging of four items into two. A pilot study with 30 healthcare professionals (face validity) was performed. Minor modifications were made based on participant feedback, preserving the 14 items.
- Items with inter-item correlations and corrected item-total correlations (item analysis, n = 162) all items were preserved.
- Exploratory factor analysis was performed (construct validity, n = 70, randomly selected), and all items were preserved
- Confirmatory factor analysis was performed (construct validity, n = 162), and all items were preserved.
- Test–retest reliability (n = 30) and Cronbach’s alpha (n = 162) were assessed. The internal consistency reliability of the final instrument was evaluated using Cronbach’s alpha (α) coefficient. Internal consistency ranged from good to acceptable (implementation α = 0.800; agreement α = 0.711). A value of α ≥ 0.70 was considered acceptable, indicating a satisfactory level of reliability for each domain of the questionnaire [24]. The reliability analysis confirmed that the instrument maintained good internal consistency across all major dimensions (care characteristics, communication, information, and continuity of care).

Moreover, in order to answer the research question, additional independent questions were incorporated based on previous studies [25,26,27,28,29]. More specifically, the following were considered:

- Independent items regarding the assessment of children and caregivers’ information (2 items) were assessed as independent variables in a 5-point Likert scale.
- Independent items capturing healthcare professionals’ views regarding the level of children’s and caregivers’ needs; the organization, safety, and overall quality of the provided care; and their personal satisfaction from their work environment (6 items) were assessed as independent variables in a 0–100 scale.
- An open-ended question was included regarding the major barriers of everyday clinical practice.

The final questionnaire provided a holistic, validated, and context-sensitive measure of pediatric healthcare quality, addressing both patient experience and professional assessment.

### 2.3. Participants and Inclusion/Exclusion Criteria

A convenience sample of 162 healthcare professionals participated in the study, including 122 pediatric doctors (75.3%), 24 nurses (14.8%), and 16 assistant nurses (9.9%) working at the emergency departments and the outpatient clinics of the two pediatric hospitals. The participants were recruited in a one-year period (November 2022–November 2023) in order to represent a range of professional backgrounds and years of experience in pediatric care. Originally, all the health professionals working in the ED of the two hospitals were invited to participate in the study. From the 198 health professionals, 30 responded to the pilot study and 168 to the main study. In six cases, the completion was partial and therefore not analyzed. The study finally included 162 responses, which indicates a participation rate equal to 96.97%.

Participants were included in the study if they met the following criteria:

- Provision of informed consent prior to participation.
- Working at the emergency department or regular pediatric outpatient clinics during the study period.
- Availability to complete the study questionnaire.

Participants were recruited during their regular work shifts. Participation was voluntary, and confidentiality and anonymity were ensured throughout the research process.

### 2.4. Recruitment and Data Collection

The principal researcher individually explained the goals and the design of the study to each eligible member of the medical and nursing staff. After providing their informed consent, participants were given a questionnaire and detailed instructions for completing it. The researcher was available to respond to questions and provide guidance as needed during the data collection period.

The participants were provided with enough time to complete the questionnaire whenever it was most convenient for them. Every stage of the study was entirely voluntary, and participants could withdraw at any moment without providing a reason or facing any consequences. During routine shifts at the emergency and outpatient departments, the researcher personally distributed the questionnaires, which were then returned in sealed envelopes to maintain confidentiality.

### 2.5. Data Analysis

Data were analyzed using descriptive statistics (means and standard deviations (for numbers) or frequencies and percentages (for categorical and dichotomous variables)). The normality of distribution for continuous variables was tested using the Shapiro–Wilk test, and inferential tests (chi-square and one-way ANOVA) were performed to identify differences among professional groups. Statistical significance was set at *p* < 0.05. Data analysis was performed using the Statistical Package for the Social Sciences, version 23.0 (SPSS, IBM Corp., Armonk, NY, USA).

### 2.6. Ethical Considerations

This study received approval from the Ethics Committee of the University of West Attica (Approval N. 110430 on 11 November 2022) along with the Scientific Councils of both hospitals. Approval was obtained from Pentelis Children’s Hospital (Approval N. 7279 on 6 April 2022) and Panagiotis and Aglaia Kyriakou Children’s Hospital (Approval N. 233 on 5 July 2022) according to institutional procedures. Data collection followed GDPR (Regulation (EU) 2016/679) and national data protection laws.

Data was collected and secured with the use of anonymous coded forms, and was accessible only to research team members. The data storage period was set at five years, under the supervision of the principal investigator. At every stage, participants’ rights to anonymity, privacy, and voluntary withdrawal were ensured. National and international guidelines for research involving human participants were strictly followed to ensure that nobody outside the research team could identify personal data.

## 3. Results

### 3.1. Demographics

A total of 162 healthcare professionals participated in the study, including 122 physicians, 24 nurses, and 16 nursing assistants. Participants were employed in the Emergency Departments of two public pediatric hospitals in Athens: “Panagiotis & Aglaia Kyriakou” (N = 93, 57.4%) and “Penteli General Children’s Hospital” (N = 69, 42.6%). The majority of participants were unmarried (N = 69, 42.6%) and without children (N = 95, 58.6%). Most worked frequent or nearly fixed shifts on a rotating schedule (N = 122, 75.3%). The majority were university graduates (N = 136, 84%), with 37% (N = 60) with master’s degrees and 11.1% (N = 18) with PhD-level education. Table 1 presents the demographic characteristics of the participants.

The participants’ mean age was 36.07 ± 9.85 years. The age distribution by sex showed that females had a wider age range: 53 females were under 30 years, 38 between 31 and 40 years, 15 between 41 and 50 years, and 11 between 51 and 60 years. Male participants included 14 under 30 years, 15 between 31 and 40 years, 8 between 41 and 50 years, 5 between 51 and 60 years, and 2 over 60 years. Overall, the <30 and 31–40 age groups comprised the majority of the sample, with 67 and 53 participants, respectively. The mean total professional experience was 10.17 ± 9.49 years, ranging from 0.2 to 40 years, and the mean employment period at the pediatric hospital was 5.06 ± 6.64 years with an average period of employment in the specific department of 4.11 ± 5.53 years.

### 3.2. Assessment of Quality of Offered Services

Healthcare professionals participating in the study responded to the 14 questions regarding the quality of offered services, indicating their level of agreement on each item as well as the degree to which these aspects were implemented in practice at their hospital. The reliability of this questionnaire section (14 questions) was assessed by calculating Cronbach’s alpha coefficient. Specifically, the internal consistency for the professionals’ agreement was α = 0.711, and for the implementation in clinical practice, it was α = 0.800, indicating good reliability for both groups of questions. The participants’ responses for each question are presented in detail in Table 2. We analyzed whether there were statistically significant associations between demographic variables and the individual items of the scale, and only the significant results are presented.

Participants’ responses to most questions did not differ significantly based on sex. The only exception concerned the adequacy of information provided to children when their cognitive level allows it, where female participants were more likely to agree that this practice is implemented (χ^2^ = 9.458, df = 4, *p* = 0.050).

Significant differences were observed based on the age of the professionals. Younger participants reported more frequently that discrimination occurs in practice based on race, religion, or nationality (χ^2^ = 23.34, df = 12, *p* = 0.025); that waiting times are longer (χ^2^ = 35.979, df = 16, *p* = 0.003); and that the approach toward pediatric patients is not always characterized by expected politeness, respect, and dignity (χ^2^ = 24.863, df = 12, *p* = 0.015). Professionals with fewer years of hospital experience also reported more frequent discrimination (χ^2^ = 21.990, df = 12, *p* = 0.038) and stressed the significance of reducing waiting times (χ^2^ = 27.160, df = 16, *p* = 0.040).

Perceptions of waiting times varied significantly depending on the hospital of origin. Professionals from Pentelis Hospital reported fewer delays than those from P & A Kyriakou Hospital (χ^2^ = 11.547, df = 4, *p* = 0.021). In particular, the majority of respondents (40 out of 69) said that waiting times at Pentelis Hospital are “sometimes” adequately prompt, whereas fewer said “sometimes” or “almost always” (16 and 3, respectively). Responses at P & A Kyriakou primarily concentrated around “rarely” and “sometimes” (30 and 48 out of 93, respectively), suggesting that staff members at both institutions thought delays were a common problem.

Younger staff members agreed more strongly that there should not be long delays and extended waiting times (χ^2^ = 27.160, df = 16, *p* = 0.040). Further analysis examined differences based on the hospital of employment. Statistically significant differences were found regarding waiting times and whether processes are carried out without delay in practice (χ^2^ = 11.547, df = 4, *p* = 0.021). Besides age and hospital, statistically significant differences regarding perceptions of waiting times and delays were found based on educational level (χ^2^ = 45.502, df = 24, *p* = 0.005), parental status (χ^2^ = 13.055, df = 4, *p* = 0.011), total years working in the department (χ^2^ = 43.048, df = 16, *p* = 0.001), and total years working at the hospital (χ^2^ = 32.396, df = 16, *p* = 0.050). Moreover, educational level was a significant factor affecting many parameters. Professionals with higher education levels strongly emphasized the need for a child-friendly environment in emergency departments and outpatient clinics (χ^2^ = 58.837, df = 24, *p* = 0.001) and recognized the importance of providing sufficient information to parents (χ^2^ = 39.092, df = 18, *p* = 0.003). However, they were also more critical regarding the protection of children’s privacy (χ^2^ = 36.476, df = 24, *p* = 0.049) and the effective management of pain (χ^2^ = 43.002, df = 24, *p* = 0.010).

Accessibility free from discrimination due to religion, race, or other social factors was identified by all participants as a priority. Younger healthcare professionals agreed more strongly (χ^2^ = 30.368, df = 12, *p* = 0.001) but reported more frequent discrimination in practice (χ^2^ = 23.34, df = 12, *p* = 0.009), a finding confirmed by those with less clinical experience (χ^2^ = 21.990, df = 12, *p* = 0.038).

Professionals with higher educational levels significantly recognized the need for children to remain in a child-friendly environment (χ^2^ = 58.837, df = 24, *p* = 0.001). Significant differences were found in practice implementation by hospital (χ^2^ = 18.943, df = 4, *p* = 0.001), educational level (χ^2^ = 56.116, df = 24, *p* = 0.001), profession (χ^2^ = 19.464, df = 8, *p* = 0.013), and years working in the department (χ^2^ = 58.837, df = 16, *p* = 0.001). Participants with higher education levels agreed more that information provided to parents regarding the child’s health status, potential diagnosis, treatments, and procedures should be adequate (χ^2^ = 39.092, df = 18, *p* = 0.003). They were also more skeptical about the actual implementation of privacy protection when providing information about a child (χ^2^ = 36.476, df = 24, *p* = 0.049).

Healthcare professionals with lower education levels showed less agreement on minimizing pain and effectively managing it (χ^2^ = 37.245, df = 18, *p* = 0.005), whereas those with postgraduate and doctoral degrees believed this was less frequently applied in practice (χ^2^ = 43.002, df = 24, *p* = 0.010). Significant differences were noted regarding the use of distraction techniques during painful procedures according to profession. Professionals with less overall experience agreed more strongly that healthcare staff should respond to the emotional concerns of children and accompanying caregivers (χ^2^ = 56.104, df = 16, *p* = 0.001). Educational level influenced perceptions of how this is implemented in practice (χ^2^ = 36.326, df = 24, *p* = 0.050). Minimizing and effectively managing pain through prevention, avoidance, or mitigation techniques was almost unanimously recognized as a priority by healthcare professionals. Differences in perceived implementation in practice were associated with profession (χ^2^ = 17.703, df = 8, *p* = 0.024), department (χ^2^ = 29.338, df = 12, *p* = 0.004), and rotating shifts (χ^2^ = 48.456, df = 16, *p* = 0.001).

Differences were also noted based on professional role. Healthcare professionals from different specialties showed varied opinions on the application of distraction techniques during painful interventions (χ^2^ = 14.219, df = 6, *p* = 0.027) and the provision of clear written discharge instructions (χ^2^ = 24.913, df = 8, *p* = 0.002). Furthermore, those working rotating shifts reported differing perceptions on communication adequacy and information delivery (χ^2^ = 30.951, df = 12, *p* = 0.002).

The health professionals were then asked to evaluate on a 5-point Likert-type scale the level of information of the parents (caregivers) and the children themselves. The detailed responses are presented in Table 3.

The last section of the study included six questions on a scale of 0–100 regarding the coverage of the needs of parents and children overall, the quality of the services provided, the degree of organization of the services, the degree of safety of the care provided in their workplace, and the satisfaction of health professionals with their work. The participants’ answers are illustrated in Table 4.

Overall need fulfillment for children (physical, emotional, psychosocial, informational, etc.) scored a mean of 61.79 (SD 15.61), with a median of 60 (range 20–100). Parents’ needs were rated slightly higher (mean 65.77, SD 17.01). Quality of hospital services received a mean score of 65.25 (SD 16.79), and satisfaction with care organization was lower (mean 60.85, SD 18.76). Safety of care was the highest-rated aspect (mean 73.27, SD 16.78), while job satisfaction was moderately high (mean 68.15, SD 19.70) but with wide variability (range 0–100). Significantly, 46.9% of participants said they were “sometimes” and 16% said they were “almost always” physically or mentally weary from their regular work, indicating a stressful work environment.

**Figure 1:**
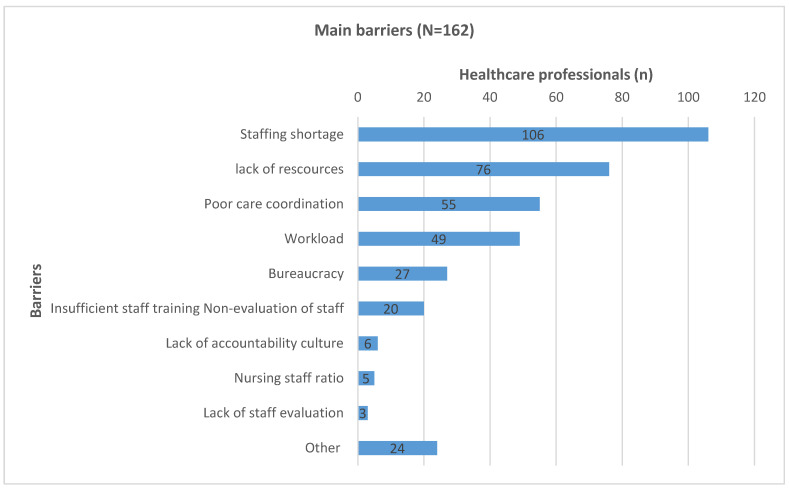
Main barriers to the provision of high-quality pediatric clinical care.

The open-ended question regarding the main barriers to everyday clinical practice was analyzed by identifying the reported barriers in the responses, which were coded through a thematic content analysis. The primary researcher and another member of the research team independently coded the barriers, a preliminary codebook was created, discrepancies were discussed and resolved by consensus, and the codebook was refined accordingly. Codes with similar meaning were grouped into higher-order categories using an inductive approach. Categories were revised until they were internally coherent and distinct from one another. When appropriate, closely related categories were combined, and overarching themes were named to reflect the shared concept. It must be noted that each response may include more than one barrier. Frequencies of codes/categories were then calculated to summarize how commonly each barrier was reported. Main barriers to everyday clinical practice were stated by 92% (N = 149) of the participants. Lack of resources (46.9%), poor coordination/organization of healthcare services (34%), workload (30.2%), inadequate staff training (12.3%), increased bureaucracy (16.7%), reluctance to take responsibility (3.7%), poor nurse-to-patient ratios (3.1%), and lack of staff evaluation (1.9%) were the primary challenges noted. Other barriers (14.8%) involved issues such as lack of primary healthcare, low socioeconomic status of parents, and communication difficulties. See Figure 1.

## 4. Discussion

The study findings present the views of healthcare professionals working in the pediatric emergency care context in two core public pediatric hospitals in Greece. The participants provided their views regarding the core values and the quality of the provided family- and child-centered care, while simultaneously describing weaker and less consistent implementation in everyday practice [29,30]. Across domains, endorsement was particularly high for non-discrimination, respectful and developmentally appropriate communication, provision of adequate information, hygiene standards, and pain minimization; however, implementation ratings were commonly lower, indicating a persistent “principle–practice” gap [1,29,31]. This pattern is consistent with international perspectives that frame pediatric EDs as settings where standards of child-centeredness are widely accepted, yet operational constraints frequently limit their reliable enactment, especially under high demand and time pressure; however, the feasibility of measurements with quality of care indicators is still questionable [1,20,31].

Among all dimensions assessed, the largest discrepancy between agreement and implementation concerned waiting times, which emerged as the most prominent perceived weakness. Although the vast majority of respondents agreed that delays should be avoided, implementation was most often rated only as “sometimes” adequate, with very few reporting that timely processes occur “almost always.” This finding reinforces the centrality of waiting time as a quality indicator in emergency care and is particularly consequential in pediatrics, where prolonged delays are associated with increased child and caregiver anxiety and affect perceived safety, trust, and overall satisfaction [28,32,33].

According to the findings of this study, participants reported limited resources and substantial gaps regarding the child-friendly environment that is acknowledged as important in emergency settings in order to enhance trust relationships with children and their caregivers, as well as provide the privacy that is recommended, especially during communication and information provision. These results suggest that psychosocial and rights-based aspects of care are especially vulnerable to infrastructure limitations, crowding, and workflow demands. The difficulty in achieving a child-friendly environment aligns with evidence that infrastructure and funding often lag behind recommendations, despite the documented benefits of child-appropriate spaces in reducing stress and improving cooperation [34,35,36]. Moreover, in line with our findings, privacy constraints are commonly described in high-volume emergency departments, where limited space and competing priorities can undermine confidential and respectful communication, particularly for older children whose autonomy and voice should be actively supported [37,38].

Additionally, gaps in everyday practice were not identified by all participants since perceptions varied by age, years of clinical experience, hospital of employment, education level, profession, department, and rotating shifts. Younger professionals and those with fewer years of experience were more likely to report problems in everyday clinical practice, longer waiting times, and less child- or family-friendly treatment, despite strong endorsement of these standards [39]. This may reflect higher sensitivity among younger professionals to indicators of equity and rights-based issues, alongside a lower tolerance for organizational shortcomings that affect the experiences of both children and their caregivers [39,40]. Unnecessary overcrowding in the ED not only increases the waiting time but also increases complaints and may progress to assault towards health professionals, especially younger ones among physicians and nurses among other health professionals [40]. In these circumstances, the relationship between a health professional and a pediatric patient or caregiver may be poor, making it difficult for healthcare professionals to provide respectful and sensitive care, and this may increase the risk of errors and unintended harm. In addition, waiting room experience, comprehensiveness of health assessments, and observations of safety measures are important for parents but seem to be underestimated by healthcare professionals [41].

Variations based on experience can also be interpreted as an “exposure” effect: extended exposure to high-load systems may increase awareness of structural flaws and persistent workflow obstacles that compromise optimal clinical practice [29]. The finding that hospital of employment affected professionals’ perceptions, especially regarding prolonged waiting time, with the staff at “P. & A. Kyriakou” reporting more frequent delays than those at Pentelis hospital, implies that patient volume, organizational procedures, bureaucracy, and local operational conditions significantly influence front-line experience. This outcome is in accordance with the larger body of research showing that ED traffic and timeliness are very sensitive to staffing models, case mix, and coordination procedures, even though the current data do not pinpoint the causative mechanisms underlying inter-hospital variations [39,40,42].

Among the professionals’ demographics, educational level emerged as a key factor across several care dimensions. Respondents with a higher education level more strongly emphasized the importance of a child-friendly environment and adequate caregiver information but tended to evaluate implementation of privacy protection and pain management more critically. This pattern may indicate that advanced training increases familiarity with formal standards and evidence-based expectations, thereby raising the benchmark against which daily practice is assessed. Such an interpretation is compatible with findings that training and professional development strengthen awareness of family and child-centered care requirements, communication quality, and supportive care practices, while simultaneously increasing recognition of deviations from best practices in constrained environments. The key to improving care quality is thus the implementation of family and child-centered approaches [43,44].

Sex differences were minimal overall, with one notable exception: female health professionals reported higher implementation regarding adequate information provision to children when cognitively appropriate. While the present study was unable to determine whether this reflects differences in role assignment, communication style, or other contextual factors, it is consistent with the general emphasis in the pediatric care literature on interpersonal communication as a modifiable contributor to quality and experience [5,27,45].

Our findings regarding information provision enhance the perception that implementation in clinical practice may be uneven. Caregivers were generally viewed as “quite informed,” whereas children were most often rated only as “moderately informed,” implying that direct child-focused communication, tailored to developmental level, may be less consistently delivered than caregiver-directed explanations. This can partly explain why children’s experiences differ from those of their caregivers [16]. Furthermore, children’s views support that familiarity with professionals, trusting relationships, and a person-centered approach contribute to reducing perceived power imbalances and increasing their confidence to engage [46].

In the literature, it is well evidenced that developmentally appropriate information and emotional support can reduce fear, improve cooperation, and strengthen the child’s sense of control, yet require time, training, and a supportive organizational culture, resources that are often scarce in emergency contexts. The same operational pressures may also contribute to variability in privacy protection during information-giving, especially when space is limited and patient throughput is prioritized [27,47].

Overall care evaluations provided a convergent picture since participants rated overall coverage of children’s and parents’ needs and service quality as moderate (mid-60s on 0–100 scales), with organization somewhat lower and safety highest (mean ~73). The relatively stronger safety ratings align with international frameworks that place safety at the center of quality assessment [29,48]. Nonetheless, comparatively lower organization ratings are noteworthy because organizational performance and coordination are upstream determinants of waiting time, information quality, and the capacity to deliver supportive care. In this respect, the concurrent reporting of moderately high but variable job satisfaction, alongside frequent physical or mental exhaustion, suggests a workforce operating under sustained strain, an issue of practical relevance given evidence that fatigue and shift work can adversely affect communication quality, care behaviors, and decision-making [49,50,51]. In a previous study in Greece with health professionals in intensive care provision, participants stated that the death of a child during a shift may alter their attitude and clinical performance due to compassion fatigue [52].

According to these trends, personnel shortages (mostly nursing), lack of resources, and inadequate coordination and organization were the most commonly mentioned barriers to the provision of the best possible care for children, followed by workload and bureaucracy. The interpretation that observed gaps are structurally driven rather than primarily attitudinal is supported by the close mapping of these perceived constraints onto the care domains with the weakest implementation, particularly delays, limited child-friendly infrastructure, and inconsistencies in privacy and supportive care.

This alignment is highly consistent with evidence from around the world showing that insufficient staffing and resources impair the flow and efficiency of emergency departments and may jeopardize patient safety and experience, while deficiencies in infrastructure and diagnostic/logistical support can impede prompt decision-making and the provision of high-quality care. The interconnected obstacles of workload, bureaucracy, and organization further suggest that efforts to improve quality should go beyond individual training and focus on system-level redesign, including standardized procedures, improved interprofessional coordination, and institutionalized mechanisms for evaluation and feedback. To enhance pediatric emergency treatment, research networks and quality improvement programs are crucial [53,54,55,56,57].

The study findings indicate that the participants greatly support child-centered standards, but face recurrent operational and environmental constraints that limit consistent implementation, most notably regarding timeliness, child-friendly spaces, and privacy in communication. These results support a dual improvement focus aiming at structural and workflow interventions to reduce delays and improve coordination, and the implementation of targeted, feasible strategies to protect child-centered communication practices. Such an approach is congruent with WHO guidance emphasizing child rights, effective communication, supportive environments, and system readiness as inseparable components of quality pediatric emergency care [27].

### 4.1. Study Limitations

Several limitations should be considered when interpreting these findings. This study relies on professionals’ self-reported perceptions rather than direct observation of clinical encounters or objective service indicators and does not include children’s and caregivers’ perspectives. Consequently, responses may reflect subjective experience, cultural context, local expectations, and subjective dissatisfaction or recent critical events rather than stable organizational patterns. The cross-sectional design captures perceptions at a single point in time, limiting causal inference regarding observed associations. This study also employed a newly developed scale that has not been applied before. The measures assess perceived “implementation” at a general level and may be affected by recall bias and the salience of recent events (for example an unusually crowded shift). Additionally, convenience sampling was employed, limited to two public hospitals and only from specific departments, which constrains generalizability to other pediatric emergency settings, with different staffing models, infrastructure, or patient case mixes. Moreover, although multiple demographic and professional variables were examined, unmeasured confounding is likely. Another important limitation was the low number of nurses and assistant nurses that participated in the study due to the tremendous lack of nursing personnel in the Greek hospitals. Therefore, the greater participation by physicians may have also shaped the overall pattern of findings.

### 4.2. Implications for Clinical Practice

The results have several important implications for pediatric emergency and outpatient care. More specifically, there is a clear need to

- Strengthen workforce capacity and address staffing shortages, especially nursing shortages.
- Improve organizational readiness and patient flow to effectively manage delays and overcrowding.
- Enhance child-friendly environments to reduce anxiety and improve the overall care experience for children and families, especially during long waiting periods.
- Standardize communication and information delivery to promote consistency and quality across professional groups and shifts.
- Implement protocol-driven procedures, adequate staff training, and sufficient resources to manage pain and suffering.
- Promote the professional growth and well-being of employees by controlling workloads, granting access to continuing education, and establishing chances for multidisciplinary cooperation.

### 4.3. Future Research Directions

In the present study, the Quality of Care Assessment Scale is used primarily as a descriptive diagnostic instrument to address professionals’ perceptions of ideal standards (agreement) and perceived delivery (implementation), enabling identification of priority gaps for quality improvement rather than benchmarking between organizations. While the tool may support longitudinal evaluation within services, comparisons across settings require additional evidence and further measurement invariance.

Future research could triangulate professionals’ perceptions with objective service indicators that are now widely implemented in Greece, along with parental and children views. Moreover, in-depth qualitative work to explain the “principle–practice gap” with interviews with physicians, nurses, assistant nurses, and managers to uncover workflow obstacles, privacy barriers, and communication constraints is strongly recommended. The identified perception-based findings may be incorporated into a broader framework that incorporates measurable indicators of care complexity since it can be challenging to assess the complexity of care in pediatric settings [58]. There is increasing evidence demonstrating that nursing care complexity indicators can anticipate organizational pressures, intra-hospital dynamics, and adverse outcomes in pediatric settings [58,59]. Therefore, there is a clear rationale for future studies aimed at integrating professionals’ perceptions with standardized care data and outcome-oriented metrics.

In addition, a wider representative sample from private and public emergency departments in Greece will confirm the preliminary validation of the Quality of Care Assessment Scale and assess measurement invariance across different settings. Finally, the study will be replicated across other pediatric centers and departments to determine whether the barriers are systemic or site-specific.

## 5. Conclusions

This study offers a comprehensive summary of how healthcare professionals assess the quality of pediatric emergency and outpatient care in two significant public children’s hospitals in Athens. The fundamental principles of high-quality family- and child-centered pediatric care—such as equitable access, respectful treatment, good communication with children and their parents, pain prevention and management, and safety—were generally well-supported by the participants. These results show that pediatric healthcare providers have a high degree of ethical commitment and professional awareness.

However, an apparent gap arose between what professionals thought should be delivered and what they see as being carried out in everyday practice. Waiting times, overcrowding, insufficient staffing, particularly nursing shortages, and inadequate resources were highlighted as the primary barriers to providing excellent services. Child-friendly infrastructure, privacy during examinations and information communication, and systematic pain treatment were considered as areas where execution was insufficient, even though these characteristics have been widely accepted as vital. Differences in perceptions based on age, education level, profession, and hospital underline the uneven impact of organizational conditions and resource availability among settings and staff groups.

The results show a high sense of safety but a moderate level of overall care quality, with significant deficiencies in waiting time management, organization, and complete psychosocial support. The need to improve family- and child-centered practices and organizational procedures to match high safety perceptions with overall quality and happiness is highlighted by the heterogeneity among professionals, departments, and shifts. In conclusion, despite the self-stated strong willingness of health professionals in Greek public pediatric hospitals to providing high-quality, child-centered care, organizational and structural issues prevent it from being consistently provided. To close the gap between standards and practice and improve the quality and sustainability of pediatric emergency care, targeted, system-level interventions that concentrate on staffing, organization, environment, and professional support are essential.

## Figures and Tables

**Table 1:** Participants’ demographics.

Sample Demographics (Ν = 162)		
	**Ν**	**%**
**Hospital**		
Pentelis General Pediatric Hospital	69	42.6%
P & A Kyriakou Pediatric Hospital	93	57.4%
**Sex**		
Male	44	27.2%
Female	118	72.8%
**Profession**		
Physician	122	75.3%
Nurse	24	14.8%
Assistant Nurse	16	9.9%
**Professional Title**		
Resident	74	45.7%
Medical Supervisor B grade	28	17.3%
Medical Supervisor A grade	11	6.8%
Deputy Director	4	2.5%
Medical Service Director	6	3.7%
Nursing Assistant	17	10.5%
Nurse	20	12.3%
Head of Nursing	1	0.6%
Deputy Director of Nursing	1	0.6%
**Working Department**		
General pediatric	50	30.9%
Pediatric specialty	42	25.9%
Surgical	33	20.4%
Not stated	37	22.8%
**Age (years)**		
<30	67	41.4%
31–40	53	32.7%
41–50	23	14.2%
51–60	16	9.9%
>60	3	1.9%
**Family Status**		
Married	59	36.4%
Divorced	9	5.6%
Widowed	2	1.2%
Unmarried	69	42.6%
In relationship	23	14.2%
**Number of Children in the family**		
0	95	58.6%
1	32	19.8%
2	25	15.4%
3	6	3.7%
Not stated	4	2.5%
**Educational Level**		
Institute of Vocational Training (IVT)/Technical Vocational School (TVS)	17	10.5%
Technological Educational Institution (TEI)	9	5.6%
University	58	35.8%
MSc	60	37.0%
PhD	18	11.1%
**Flexible Working Hours/Shifts**		
Almost never	7	4.3%
Rarely	3	1.9%
Sometimes	30	18.5%
Often	73	45.1%
Almost always	49	30.2%

**Table 2:** Participants’ responses per question regarding the quality of services offered and the statistically significant influencing factors per question.

Questions	Level of Agreement	Ν (%)	*p* Values *	Level of Implementation in Practice	Ν (%)	*p* Values *
Question 1. Care and access for young patients in the hospital should be provided without any discrimination based on race, religion, or ethnicity.	Totally disagree	3 (1.9%)	Age, *p* = 0.001 Family status, *p* = 0.029	Almost never/never	0 (0.0%)	Age, *p* = 0.025 Total hospital experience, *p* = 0.038
	Disagree	0 (0.0%)		Rarely	2 (1.2%)	
	Neither agree or disagree	1 (0.6%)		Sometimes	16 (9.9%)	
	Agree	10 (6.2%)		Often	40 (24.7%)	
	Totally agree	148 (91.4%)		Almost every time/every time	104 (64.2%)	
	Total answers	162 (100.0%)		Total answers	162 (100.0%)	
Question 2. Waiting times for procedures concerning health issues or administrative matters related to the pediatric patient should be sufficient and carried out in such a way that there is no delay.	Totally disagree	1 (0.6%)	Total experience in the department, *p* = 0.040	Almost never/never	4 (2.5%)	Age, *p* = 0.003 Hospital, *p* = 0.021 Education, *p* = 0.005 Children (yes/no), *p* = 0.011 Total years of experience in hospital, *p* = 0.009 Total experience in the department, *p* = 0.001
	Disagree	3 (1.9%)		Rarely	38 (23.5%)	
	Neither agree or disagree	6 (3.7%)		Sometimes	88 (54.3%)	
	Agree	43 (26.5%)		Often	26 (16.0%)	
	Totally agree	109 (67.3%)		Almost every time/every time	6 (3.7%)	
	Total answers	162 (100.0%)		Total answers	162 (100.0%)	
Question 3. Both pediatric patients and their caregivers should be approached in a polite manner and all cases should be treated with respect and dignity.	Totally disagree	0 (0.0%)		Almost never/never	0 (0.0%)	Age, *p* = 0.015 Total hospital experience, *p* = 0.019
	Disagree	0 (0.0%)		Rarely	2 (1.2%)	
	Neither agree or disagree	5 (3.1%)		Sometimes	40 (24.7%)	
	Agree	31 (19.1%)		Often	80 (49.4%)	
	Totally agree	126 (77.8%)		Almost every time/every time	40 (24.7%)	
	Total answers	162 (100.0%)		Total answers	162 (100.0%)	
Question 4. The environment where children stay in the Emergency Department/Outpatient Clinics should be child-friendly. There should be a playroom equipped with books and toys appropriate for each age.	Totally disagree	1 (0.6%)	Education, *p* = 0.001	Almost never/never	60 (37.0%)	Hospital, *p* = 0.001 Education, *p* = 0.001 Profession, *p* = 0.013 Total experience in the department, *p* = 0.001
	Disagree	4 (2.5%)		Rarely	46 (28.4%)	
	Neither agree or disagree	15 (9.3%)		Sometimes	41 (25.3%)	
	Agree	40 (24.7%)		Often	9 (5.6%)	
	Totally agree	102 (63.0%)		Almost every time/every time	6 (3.7%)	
	Total answers	162 (100.0%)		Total answers	162 (100.0%)	
Question 5. The overall hygiene conditions, regarding the space in the Emergency Department/Outpatient Clinic of a Pediatric Hospital, should be excellent. (The ward should be clean, waste should be collected properly and all hygiene rules should be implemented.)	Totally disagree	0 (0.0%)		Almost never/never	5 (3.1%)	Education, *p* = 0.001 Total experience in the department, *p* = 0.016
	Disagree	0 (0.0%)		Rarely	22 (13.6%)	
	Neither agree or disagree	0 (0.0%)		Sometimes	52 (32.1%)	
	Agree	11 (6.8%)		Often	60 (37.0%)	
	Totally agree	151 (93.2%)		Almost every time/every time	23 (14.2%)	
	Total answers	162 (100.0%)		Total answers	162 (100.0%)	
Question 6. Health professionals should be responsive to the emotional concerns of children and their accompanying caregivers.	Totally disagree	1 (0.6%)	Total years of experience, *p* = 0.001	Almost never/never	2 (1.2%)	Education, *p* = 0.050
	Disagree	3 (1.9%)		Rarely	16 (9.9%)	
	Neither agree or disagree	15 (9.3%)		Sometimes	63 (38.9%)	
	Agree	56 (34.6%)		Often	71 (43.8%)	
	Totally agree	87 (53.7%)		Almost every time/every time	10 (6.2%)	
	Total answers	162 (100.0%)		Total answers	162 (100.0%)	
Question 7. Adequate and appropriate communication with hospitalized children and their parents (caregivers) should be ensured.	Totally disagree	0 (0.0%)		Almost never/never	0 (0.0%)	Profession, *p* = 0.027
	Disagree	0 (0.0%)		Rarely	8 (4.9%)	
	Neither agree or disagree	2 (1.2%)		Sometimes	54 (33.3%)	
	Agree	35 (21.6%)		Often	81 (50.0%)	
	Totally agree	125 (77.2%)		Almost every time/every time	19 (11.7%)	
	Total answers	162 (100.0%)		Total answers	162 (100.0%)	
Question 8. Information to parents (caregivers) of children, as well as to children, should be provided in a culturally appropriate and understandable manner.	Totally disagree	0 (0.0%)	Working in shifts, *p* = 0.002	Almost never/never	1 (0.6%)	Working in shifts, *p* = 0.035
	Disagree	2 (1.2%)		Rarely	8 (4.9%)	
	Neither agree or disagree	1 (0.6%)		Sometimes	51 (31.5%)	
	Agree	26 (16.0%)		Often	67 (41.4%)	
	Totally agree	133 (82.1%)		Almost every time/every time	35 (21.6%)	
	Total answers	162 (100.0%)		Total answers	162 (100.0%)	
Question 9. Information to children should be adequate regarding their health condition, possible diagnosis, potential treatments and procedures. (As long as Age > 4 years and their cognitive level allows it.)	Totally disagree	1 (0.6%)		Almost never/never	3 (1.9%)	Sex, *p* = 0.050 Profession, *p* = 0.013
	Disagree	5 (3.1%)		Rarely	31 (19.1%)	
	Neither agree or disagree	27 (16.7%)		Sometimes	69 (42.6%)	
	Agree	57 (35.2%)		Often	42 (25.9%)	
	Totally agree	72 (44.4%)		Almost every time/every time	17 (10.5%)	
	Total answers	162 (100.0%)		Total answers	162 (100.0%)	
Question 10. Information to parents (caregivers) should be sufficient regarding the child’s health status, possible diagnosis, potential treatments and procedures.	Totally disagree	0 (0.0%)	Profession, *p* = 0.013 Education, *p* = 0.003	Almost never/never	0 (0.0%)	Profession, *p* = 0.002 Department, *p* = 0.005
	Disagree	1 (0.6%)		Rarely	6 (3.7%)	
	Neither agree or disagree	1 (0.6%)		Sometimes	24 (14.8%)	
	Agree	21 (13.0%)		Often	70 (43.2%)	
	Totally agree	139 (85.8%)		Almost every time/every time	62 (38.3%)	
	Total answers	162 (100.0%)		Total answers	162 (100.0%)	
Question 11. The information provided to the children’s guardians upon discharge should be written and clear.	Totally disagree	0 (0.0%)		Almost never/never	4 (2.5%)	Profession, *p* = 0.002
	Disagree	0 (0.0%)		Rarely	12 (7.4%)	
	Neither agree or disagree	5 (3.1%)		Sometimes	33 (20.4%)	
	Agree	34 (21.0%)		Often	61 (37.7%)	
	Totally agree	123 (75.9%)		Almost every time/every time	52 (32.1%)	
	Total answers	162 (100.0%)		Total answers	162 (100.0%)	

* In the table are illustrated only variables with *p* ≤ 0.05.

**Table 3:** Assessment of the parental and children information levels (N = 162).

Assessment of the Parental and Children Information Level				
Likert Scale	Level of Parental Information		Level of Child Information	
	Ν	%	Ν	%
Not at all informed	0	0	10	6.2
Slightly informed	4	2.5	35	21.6
Moderately informed	48	29.6	78	48.1
Quite informed	81	50.0	27	16.7
Very informed	29	17.9	12	7.4
**Total answers**	**162**	**100.0**	**162**	**100.0**

**Table 4:** Evaluation of provided services and degree of satisfaction (N = 162).

Evaluation Parameter (Score 0–100)	Mean Value	Standard Deviation	Median	Minimum Value	Maximum Value	Intra-Quartile Range
To what extent do you believe that the overall needs (physical, emotional, psychosocial, informational, etc.) of children are covered?	61.79	15.61	60	20	100	50–70
To what extent do you believe that the overall needs (physical, emotional, psychosocial, informational, etc.) of parents/caregivers are covered?	65.77	17,01	70	10	100	58.75–80
How do you evaluate the quality of the health services provided at the hospital where you work?	65.25	16.79	70	20	100	50–80
How satisfied are you with the organization of the care provided to children and parents?	60.85	18.76	60	0	100	50–80
How safe do you consider the care provided to children and parents to be?	73.27	16.78	75	20	100	63.75–90
How satisfied are you with your work in this specific department (regular outpatient clinics)?	68.15	19.70	70	0	100	60–80

## Data Availability

The original contributions presented in this study are included in the article. Further inquiries can be directed to the corresponding author.
